# Effects of Citrus-derived Diosmetin on Melanoma: Induction of Apoptosis and Autophagy Mediated by PI3K/Akt/mTOR Pathway Inhibition

**DOI:** 10.2174/0118715206360266250115065234

**Published:** 2025-01-21

**Authors:** Jie Li, Mingyuan Xu, Nanhui Wu, Fei Wu, Jiashe Chen, Xiaoxiang Xu, Fei Tan, Yeqiang Liu

**Affiliations:** 1 Shanghai Skin Disease Clinical College, The Fifth Clinical Medical College, Anhui Medical University, Shanghai Skin Disease Hospital, Shanghai, 200443, China;; 2 Shanghai Skin Disease Hospital, Tongji University School of Medicine, Shanghai, 200443, China

**Keywords:** Diosmetin, cutaneous melanoma, autophagy, apoptosis, PI3K/Akt/mTOR, anti-tumor

## Abstract

**Background:**

Diosmetin (DIOS) is a naturally abundant flavonoid and possesses various biological activities that hold promise as an anti-cancer agent. However, the anti-cancer activities and underlying mechanism of DIOS on cutaneous melanoma remain unclear.

**Objective:**

This study seeks to explore the anti-tumor effect and mechanism of DIOS in cutaneous melanoma.

**Methods:**

Here, a variety of *in vitro* and *in vivo* experiments, combined with RNA sequencing (RNA-seq), were employed to ascertain the potential anti-cutaneous melanoma capacity and mechanism of DIOS.

**Results:**

The results demonstrated that DIOS considerably impeded cell proliferation and triggered cell apoptosis in a dose- and time-dependent manner. Concurrently, DIOS markedly elevated the expression of pro-apoptotic proteins (Cleaved caspase-3, Bax, Cleaved PARP, and Cleaved caspase-9) and downregulated the expression of Bcl-2. Additionally, DIOS markedly upregulated the protein expressions of LC3B-II and Atg5, while downregulating p62 protein expression. Notably, pre-treatment with an autophagy inhibitor significantly inhibited DIOS-induced cell apoptosis and autophagy. Mechanistically, DIOS was identified to repress the PI3K/Akt/mTOR signaling pathway by western blot analyses and RNA-seq. Finally, *in vivo* experiments using a syngeneic mouse model confirmed the anti-tumor effect of DIOS, which exhibited high levels of apoptosis and autophagy.

**Conclusion:**

These findings propose that DIOS acts as a potential melanoma therapy that exerts its anti-tumor effects by triggering apoptosis and autophagy *via* inhibition of the PI3K/Akt/mTOR pathway.

## 1 INTRODUCTION

Cutaneous melanoma, a highly aggressive skin cancer, is a malignant growth derived from melanocytes [1]. This highly malignant tumor is characterized by a distressing global rise in both incidence and mortality rates, estimated at approximately 3-5% annually [2]. There are many different approaches for treating patients with cutaneous melanoma, including surgical resection, immune checkpoint therapy, targeted therapy, and radiotherapy [3, 4]. However, the efficacy of these interventions is often limited in the advanced stages of melanoma. The propensity of tumor cells to develop resistance to chemotherapeutic agents further compounds the challenge, resulting in disappointing overall survival outcomes [5]. Consequently, the quest for novel treatment strategies that offer enhanced efficacy with reduced toxicity is both urgent and imperative. Autophagy, a cellular degradation process involving the delivery of cytoplasmic contents to lysosomes *via* double-membrane autophagosomes, plays a complex role in cancer biology [6]. While autophagy is typically regarded as a mechanism that alleviates cellular stress to support survival, its dysregulation has been implicated in the development of cancer [7]. During the initial phases of tumorigenesis, autophagy can form characteristic autophagosomes, which prevent the occurrence of tumors by inhibiting the accumulation of p62 [8]. Apoptosis, a well-known form of programmed cell death, can interact with autophagy in tumors, exhibiting either synergistic or antagonistic effects [9, 10]. In malignancies like liver and colorectal cancer [11], dysregulated activation of the PI3K/Akt/mTOR signaling pathway has been implicated in melanoma progression [12, 13]. The PI3K/Akt/mTOR pathway has been thoroughly studied in melanoma [14, 15]. As a downstream effector in the PI3K pathway, the mTOR has emerged as a critical node for regulating cellular growth and nutrient sensing, influencing key processes like survival, proliferation, autophagy, and metabolism. It is widely recognized that the PI3K/Akt/mTOR pathway acts as a negative regulator of autophagy [16].

Natural compounds have emerged as a promising avenue in cancer therapy, with numerous substances demonstrating the capacity to inhibit tumor cell growth either directly or indirectly [17]. For more than half a century, natural products with significant chemical biodiversity have been thoroughly investigated for their anticancer compounds. While flavonoids like quercetin and luteolin have been extensively studied for their anticancer effects [18-20], DIOS remains underexplored, making it a unique candidate for investigation in the context of melanoma therapy. Diosmetin (DIOS), a flavonoid commonly found in chrysanthemums, olives, and citrus fruits [21], demonstrates various biological effects, notably its antioxidant, anti-inflammatory [22], and tumor-suppressing activities. Its potential in cancer therapeutics is well-documented, with research highlighting its efficacy against liver, esophageal, and breast cancers [23, 24]. It is the main mechanism of the anti-cancer effects of DIOS that inhibits cell proliferation, promotes apoptosis, restricts tumor cell migration and sensitizes the response to chemotherapy and radiotherapy [25]. These anti-tumor activities are linked to the suppression of key signaling pathways, such as Nrf2 and Transforming Growth Factor β (TGF-β), as well as the TGF-β/protein kinase C/Mitogen-Activated Protein Kinase/Matrix Metalloproteinases (MAPK/MMP) pathway [24, 26, 27]. Additionally, DIOS has been reported to suppress cutaneous melanoma growth and angiogenesis [28]. However, the underlying mechanisms of DIOS’s therapeutic effects remain to be fully elucidated, and the inhibitory impact of DIOS on melanoma has yet to be extensively explored. Consequently, elucidating the mechanisms by which DIOS exerts its effects on cutaneous melanoma is of significant importance and urgency.

This study seeks to explore the anti-tumor effect and mechanism of DIOS in cutaneous melanoma. The impact of DIOS on cell proliferation, apoptosis, and migration was examined *in vitro*. *In vivo* experiments in mice provided evidence that DIOS exerts an inhibitory effect on melanoma and is relatively low toxic. RNA sequencing (RNA-seq) and Western blot were then used to investigate the potential anti-tumor mechanism. Results showed that DIOS exerts prominent anti-tumor capacity, significantly inhibiting cell proliferation and migration and promoting apoptosis and autophagy. Our finding would provide an experimental foundation for the potential use of DIOS in treating cutaneous melanoma.

## 2 MATERIALS AND METHODS

### 2.1 Chemicals and Reagents

**Fig. (1):**
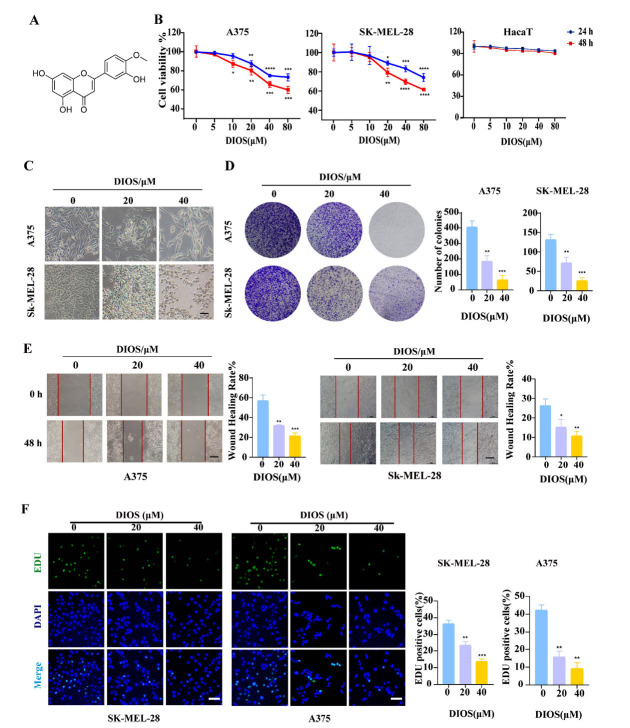
DIOS inhibits the proliferation of A375 and SK-MEL-28 cells *in vitro*. (**A**) Chemical structure of Diosmetin (DIOS). (**B**) Effect of DIOS on cell viability. (**C**) Effect of DIOS on cell morphological change (magnification, 20×). (**D**) Effect of DIOS on cell clonogenesis capacity. (**E**) Effect of DIOS on cell migration and quantitation of cell migration area. (**F**) Effect of DIOS on cell proliferation detected by EdU assay. * *p* < 0.05, ** *p* < 0.01, *** *p* < 0.001 *vs*. the control group.

Diosmetin (DIOS, 98% purity) was purified and sourced from Shanghai Macklin Biochemical Technology Co., Ltd. (Fig. **1A**). The initial dissolution of 10 mg of DIOS in 2.2203 mL of dimethyl sulfoxide (DMSO, Beyotime, China) yielded a 0.015 M solution, which was subsequently diluted with DMEM medium (Biological Industries, Haoze Biotechnology Co., Ltd, China) to attain the requisite experimental concentrations (DMSO concentrations were carefully controlled to remain below 0.1% *in vitro*, the concentration *in vivo* below 5%). The autophagy inhibitor 3-Methyladenine (3-MA) was sourced from Abmole (M2296, USA).

### 2.2 Cell Culture and Treatments

A375 and SK-MEL-28 melanoma cells (human), B16F10 melanoma cells (mouse), and HaCaT epidermal cells (human) were obtained from the Cell Bank of the Chinese Academy of Sciences in Shanghai, China. These cells were maintained in DMEM or RPMI-1640 medium (Thermo Fisher Scientific, Yingwei Jieji Trading Co., Ltd, China), supplemented with 10% fetal bovine serum (FBS, bioexplorer, Shanghai YuanChuang Biotechnology Co., Ltd, China) and 100 U/mL of streptomycin and penicillin (VivaCell, Shanghai XP Biomed Ltd., China). Standard culturing of the cells was carried out at 37°C in a 5% CO_2_ incubator (Thermal Fisher Inc, USA).

### 2.3 Cell Viability Assay

The CCK-8 assay (Vazyme, China) was employed to evaluate the effect of DIOS (0–80 μM) on cell viability. The cells were seeded in a 96-well plate at a cellular density of 1×10^4^ cells/well and treated with DIOS for a period of 48 hours. After treatments, 10 μL of CCK-8 was added and maintained for a 30-minute incubation period [28], then the absorbance was quantified, followed by absorbance measurement with a microplate reader (BIOTEK, USA).

### 2.4 Colony Formation Assay

After seeding 1 × 10^3^ cells per well in 6-well plates, DIOS (0, 20, 40 μM) was added, and the cells were incubated for 48 hours. They were then fixed in 4% paraformaldehyde for 30 minutes and washed with PBS. After a 20-minute staining period with a 0.1% crystal violet solution, the colonies were photographed following PBS washing [23].

### 2.5 Cell Wound Scratch Assay

Cells (2 × 10^4^) were seeded in 6-well plates with media containing 10% FBS. A uniform scratch was created using a sterile pipette tip with an approximate width of 1 mm [7]. Cells were treated with 0, 20, and 40 μM DIOS for 48 h. Scratch closure was evaluated at 0 and 48 hours and then photographed under a microscope (MOTIC, China).

### 2.6 Live/Dead Cell Observation

Cells (8 × 10^3^) in 96-well plates were subjected to various DIOS concentrations and, incubated for 48 h, and incubated with Calcein/PI working solution (Beyotime, China). After a 30-minute growing in the dark at 37°C, the staining was examined under a fluorescence microscope, with Calcein exhibiting green fluorescence and PI exhibiting red fluorescence.

### 2.7 Annexin V-FITC/PI Apoptotic Cell Analysis

The cells were plated in 6-well plates and subsequently subjected to DIOS (0, 20, and 40 μM) for a period of 48 hours. For analysis, the cells received 10 µL of Annexin V-FITC (Beyotime, China) and 5 µL of PI, which were then incubated for 15 minutes at 37°C in darkness [29, 30].

### 2.8 EDU Cell Cycle Assay

A375 and SK-MEL-28 cells were treated with DIOS (0, 20, and 40 μM) for 48 h, followed by incubation with 10 μM 5-ethynyl-2-deoxyuridine (EdU) for 3 hours. Cells were harvested, and EdU incorporation was analyzed using the BeyoClick™ EdU Cell Proliferation Kit. Following the application of EdU reaction buffer, cells were incubated at 25°C for about 30 minutes in the dark and imaged by fluorescence microscopy (MOTIC, China).

### 2.9 Western Blot Analysis

After different treatments, protein extraction was performed by RIPA lysis buffer (Shanghai YuanChuang Biotechnology Co., Ltd, China). Subsequently, the specimens were subjected to SDS-PAGE and transferred to 0.45 µm PVDF membranes (Merck, Haoze Biotechnology Co., Ltd, China). Target proteins were detected using a panel of specific antibodies, including Bcl-2 (AF6139, Affinity), Bax (AF0083, Affinity), Cleaved-PARP (#5625, Cell Signaling Technology), Cleaved-caspase 3 (9664S, CST), Cleaved-caspase 9 (#9509, CST), LC3B (#3868, CST), Atg5 (#12994, CST), p62 (#39749, CST), p-PI3K (#AF3242, Affinity), PI3K (#4249, CST), Akt (#9272, CST), p-Akt (#4060, CST), mTOR (#2983, CST), and p-mTOR (#5536, CST). GAPDH (#5174, CST) was employed as a loading control. Protein visualization was conducted using secondary antibodies and detected *via* ECL chemiluminescence on the Tanon 4200 system.

### 2.10 Transmission Electron Microscopy and Ultrastructure Observation

In 6 cm diameter dishes, A375 cells were cultivated and treated with DIOS (0 μM and 40 μM) for 48 h. Then, cells were fixed using precooled glutaraldehyde (Shanghai Macklin Biochemical Technology Co., Ltd. China) for 1 hour, after which they were collected and centrifuged for 5 mins. After removing the supernatant, the specimens were subjected to additional fixation and transferred to 1.5 mL tubes for electron microscopy section preparation [31]. The ultrastructure was analyzed using an electron microscope (Hitachi H-7650, Japan).

### 2.11 AdPlus-mCherry-GFP-LC3B

The expression of AdPlus-mCherry-GFP-LC3B (Beyotime, China) was achieved by cloning the LC3B gene with GFP and mCherry tags into a vector with an MOI of 20. The vector was transformed into competent cells and cultured to amplify the plasmid. The plasmid was transfected into A375 and SK-MEL-28 cells using Lipofectamine. Fluorescence microscopy confirmed GFP and mCherry expression after 24 hours. Section 2.10 outlines the methods used for culturing and man-aging A375 and SK-MEL-28 cells. For autophagy analysis, fluorescence microscopy was employed to monitor LC3B localization [32].

### 2.12 RNA-Seq

A375 cells were grown and processed following the procedures outlined in section 2.10. RNA extraction was performed using a Trizol reagent (Beyotime, China). The NanoDrop 2000 spectrophotometer and Agilent Technologies 2100 Bioanalyzer (Santa Clara City, California, USA) were used to carefully assess the concentration, purity, and integrity of the RNA samples. Library construction and transcriptome sequencing were implemented by Shanghai Ouyi Biotechnology Co., Ltd. (Shanghai, China).

### 2.13 *In vivo* Study

C57BL/6 male mice (5 weeks old, 19g, No. SYXK2018-0034) were obtained from SPF Biotechnology Co., Ltd. (Jiangsu, China) and housed in a laminar air-flow space. For the *in vivo* assays, 2×10^^5^ B16F10 cells were injected into the left axilla of the mice through a subcutaneous method [33]. When the tumor volume reached 100 mm^3^, twenty mice were randomly divided into 4 groups by tumor volume. Four groups of mice were administered DIOS (0, 20, 50, or 100 mg/kg) by gavage every other day up to day 21 [23, 34]. Meanwhile, the width and length using the digital caliper tumors were palpable, and the tumor growth was under observation at three-day intervals. The tumor volume (mm^3^) =0.5 × V × v^2^, the long and shortest diameters of the tumors, are represented by ‘V’ and ‘v,’ respectively [35]. All mice were sacrificed by excessive anesthesia (5% isoflurane). A thorough dissection, weighing, and photographing of their tumor tissues was performed. Following sacrifice, viscera were stained with Hematoxylin-Eosin (HE) staining, as previously described [36], and tumors were harvested and fixed in paraformaldehyde for the following experiments.

### 2.14 TUNEL Assay

A TUNEL assay kit (Vazyme, China) was used to assess apoptosis, following the manufacturer’s protocol [37]. The presence of apoptotic cells was assessed in randomly chosen regions of the slides with a light microscope (MOTIC, China).

### 2.15 Immunohistochemistry

The tumor tissues were fixed and immunostained using antibodies against p-Akt (1:400), LC3B (1:1600), Cleaved-caspase-3 (1:200), and Ki67 (1:200) antibodies. Stained tissues were photographed using a digital slice scanner (KFBIO, China).

### 2.16 Statistical Analysis

Mean ± SD values are reported, with statistical analyses conducted *via* GraphPad Prism. Student's t-tests were employed for the comparison of two groups, while a one-way ANOVA test was used for multiple comparisons. *p* < 0.05 was viewed as statistically significant. (Supplementary Material)

## 3 RESULTS

### 3.1 DIOS Suppresses Melanoma Cell Proliferation and Migration

The effects of DIOS on the viability of A375, SK-MEL-28, and HaCaT cells are illustrated in Fig. (**1B**). Increasing DIOS concentrations led to a dose-dependent decrease in the viability of A375 and SK-MEL-28 cells (*p* < 0.05), with a more significant reduction at 48 hours compared to 24 hours. In contrast, DIOS exhibited significantly minimal cytotoxicity towards the HaCaT, with no significant reduction in viability observed (*p* > 0.05). On the basis of these results, the concentrations of 20 and 40 µM DIOS were deemed optimal for the subsequent experiments. The morphological changes of A375 and SK-MEL-28 cells induced by different DIOS concentrations are shown in Fig. (**1C**). At higher concentrations, cells became more rounded and less adherent, indicating cytotoxicity. Significant suppression of melanoma cell colony numbers was also observed with DIOS compared to the control group (*p* < 0.01) (Fig. **1D**). Additionally, DIOS impaired the migratory ability of A375 and SK-MEL-28 cells, as evidenced by a reduced wound healing area in scratch assays (*p* < 0.05) (Fig. **1E**). The results of the EdU assay corroborate the inhibitory effects of DIOS on cell migration. Both cell lines showed a concentration-dependent decrease in proliferative capacity (*p* < 0.01) (Fig. **1F**).

### 3.2 DIOS Induces Apoptosis in Melanoma Cells

**Fig. (2):**
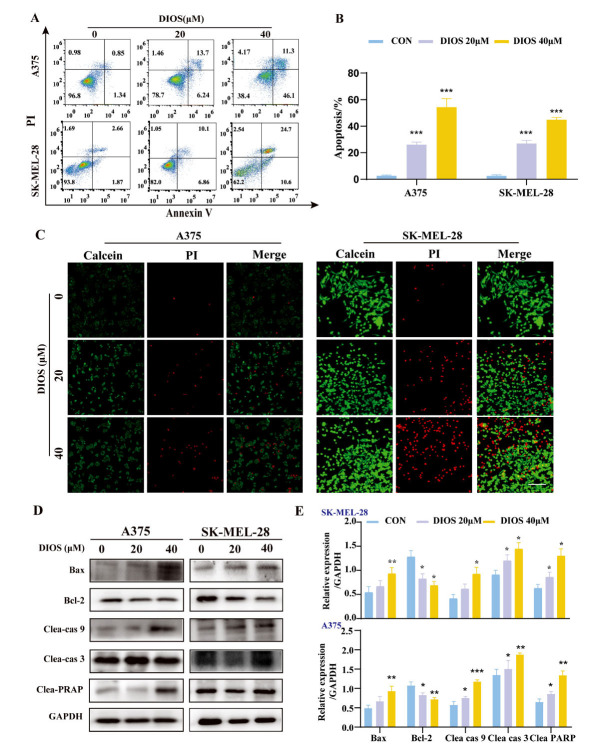
DIOS induces apoptosis in cutaneous melanoma cells *in vitro*. (**A**, **B**) Cell apoptosis evaluation of cell lines treated with DIOS by flow cytometry. (**C**) The characteristics of apoptotic nuclei in cells were observed by Calcein/PI staining. scale bar =200 μm. (**D**, **E**) The expressions of apoptotic protein. * *p* < 0.05, ** *p* < 0.01, *** *p* < 0.001 *vs*. the control group.

As depicted in Figs. (**2A**, **B**), a significant rise in both early and late apoptosis was observed. As the DIOS concentration increased, there was a considerable upsurge in the rate of apoptotic cells across both cell lines (*p* < 0.001). This was further corroborated by the Live/Dead assay, which revealed a reduction in the number of viable cells, as evidenced by the decrease in Calcein-positive cells and an increase in PI-positive cells (Fig. **2C**). Moreover, DIOS profoundly increased the levels of pro-apoptotic proteins Bax, Cleaved caspase-3, Cleaved caspase-9, and Cleaved PARP, and decreased the anti-apoptotic protein Bcl-2, as confirmed by western blot analysis (*p* < 0.05) (Figs. **2D**, **E**).

### 3.3 DIOS Promotes Autophagy in Melanoma Cells

**Fig. (3):**
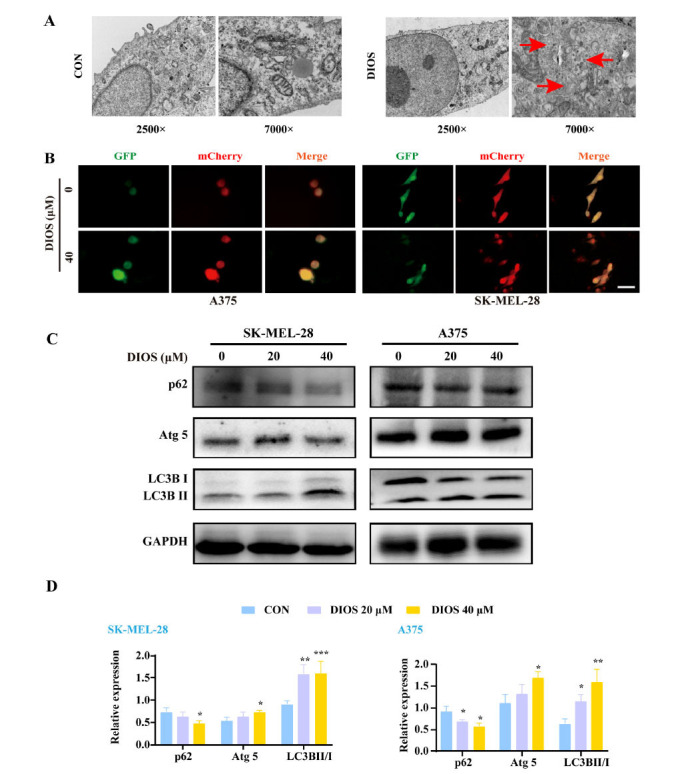
DIOS induces autophagy in melanoma cells *in vitro*. (**A**) A375 cells were exposed to 0 and 40 μM DIOS for 48 hours, and the cellular ultrastructure was examined by transmission electron microscopy (red arrow indicates autolysosome). (**B**) DIOS promoted autophagic flux in A375 and SK-MEL-28 cells. Cells were infected with 20 MOI for 24 h. Then the infected cells were treated with 40 μM DIOS for 48 h, followed by fluorescence microscopy observation. Bar = 50 μm. (**C**, **D**) The protein expressions of autophagy-related proteins p62, Atg5, and LC3B were measured. * *p* < 0.05, ** *p* < 0.01, *** *p* < 0.001 *vs*. the control group.

We utilized Transmission Electron Microscopy (TEM) to visualize the formation of autophagolysosomes. The TEM results showed that, in DIOS-treated A375 cells, the number of autophagolysosomes was significantly increased (Fig. **3A**). As shown in Fig. (**3B**), 40 μM DIOS significantly increased LC3B fluorescence intensity. Western blot analysis further confirmed that DIOS significantly increased the LC3B II/LC3B I protein ratio and Atg5 protein levels and downregulated the p62 protein expression (*p* < 0.05) (Figs. **3C**, **D**), suggesting that treatment with DIOS may be able to induce autophagy in both A375 and SK-MEL-28 cells.

### 3.4 3-MA Inhibited DIOS-induced Autophagy and Apoptosis in Melanoma Cells

**Fig. (4):**
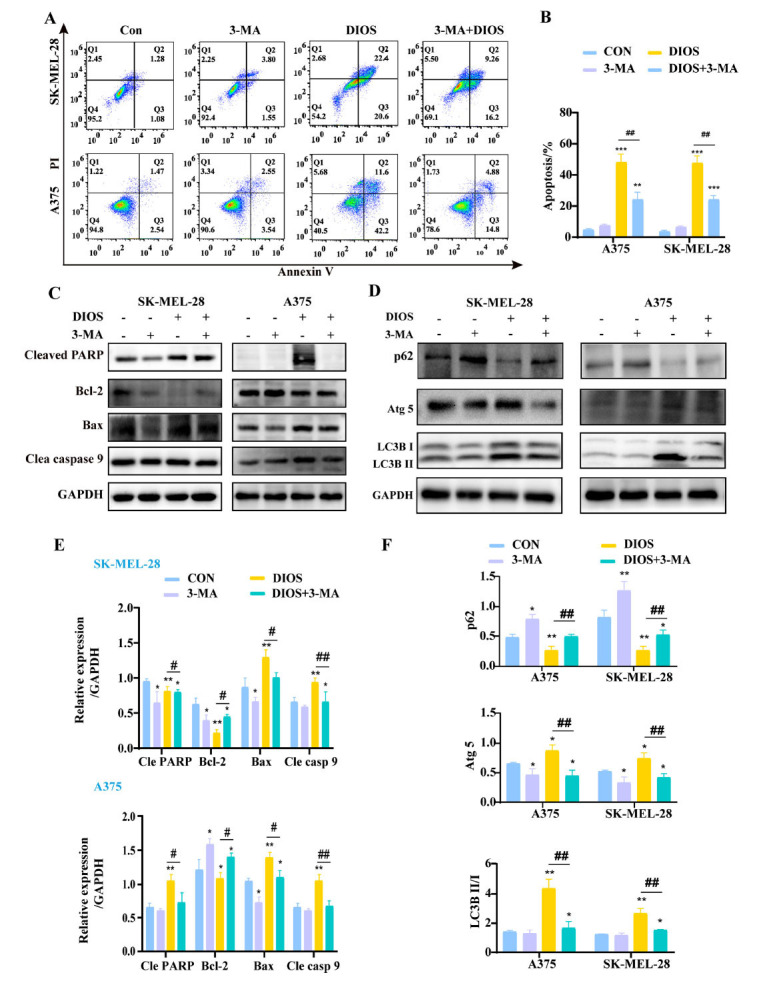
3-MA inhibits DIOS-induced apoptosis and autophagy. (**A**, **B**) Detection of apoptosis in melanoma cells treated with DIOS (40 μM) in the presence or absence of 3-MA (5 μM) by flow cytometry. (**C**, **D**) The protein expressions of p62, Atg 5, LC3B I, and LC3B II. (**E**, **F**) Quantitative analysis of these proteins. * *p* < 0.05, ** *p* < 0.01, *** *p* < 0.001 *vs*. the control group, # *p* < 0.05, ## *p* < 0.01 *vs*. the DIOS (40 μM) group.

To examine whether the induction of autophagy contributes to DIOS-induced apoptosis, the cells were pretreated with 3-MA (5 μM) for 24 h [24]. The results revealed that the group of 3-MA alone had no substantial effects on apoptosis while significantly decreasing the apoptosis rates induced by DIOS in A375 and SK-MEL-28 cells (*p* < 0.01) (Figs. **4A**, **B**). Moreover, we also observed that 3-MA markedly abrogated the induction of the pro-apoptotic protein Bax, Cleaved caspase 9, and Cleaved PARP, as well as the suppression of anti-apoptotic protein Bcl-2 expression compared with the DIOS group (*p* < 0.05) (Figs. **4C**-**F**), indicating 3-MA pretreatment could reverse DIOS-induced autophagy and apoptosis in melanoma cells.

### 3.5 DIOS Induces Melanoma Cell Apoptosis and Autophagy by Suppressing PI3K/Akt Pathway

**Fig. (5):**
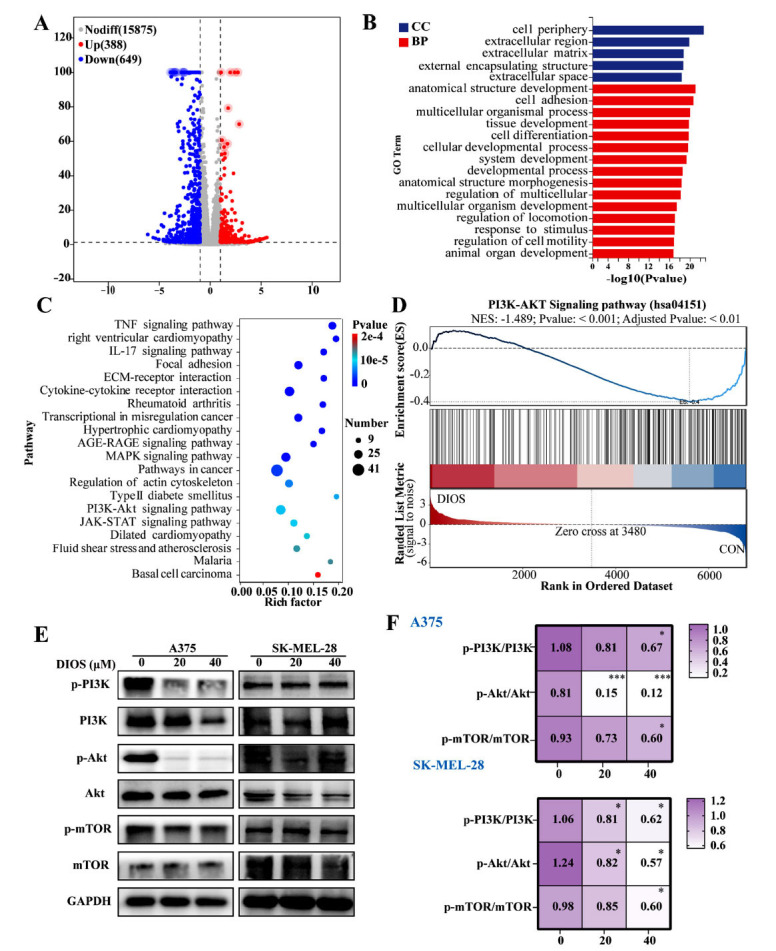
DIOS induces apoptosis and autophagy *via* the PI3K/Akt/mTOR pathway. (**A**) A375 cells were exposed to 40 µM DIOS for 48 hours and then analyzed through transcriptome sequencing. The cluster analysis heat map of down-regulated differential genes. (**B**, **C**) GO and KEGG Pathway Enrichment Analysis. (**D**) GSEA showed that the PI3K/Akt Targets pathway was mainly enriched, and DIOS significantly inhibited this pathway. (**E**, **F**) The protein expression levels of p-PI3K, PI3K, p-Akt, Akt, p-mTOR and mTOR. * *p* < 0.05, ** *p* < 0.01, *** *p* < 0.001 *vs*. the control group.

To enhance our understanding of DIOS's role in apoptosis and autophagy, RNA-seq was performed in A375 cells treated with 40 μM DIOS. Fig. (**5A**) reveals 1037 differentially expressed genes (DEGs) after DIOS administration, including 388 upregulated and 649 downregulated genes (The specific upregulated and downregulated genes are listed in the Supplementary Table). Subsequently, a GO enrichment analysis demonstrated that the DIOS treatment primarily influenced cell adhesion, system development, and cellular developmental processes (Fig. **5B**). Then, the KEGG enrichment analysis demonstrated that these DEGs were highly involved in oncogenic pathways in cancer, PI3K/Akt, MAPK, and JAK-STAT signaling pathway (Fig. **5C**). In addition, further GSEA database analysis revealed that the PI3K/Akt signaling pathway was significantly inhibited after DIOS treatment (Fig. **5D**). Furthermore, the results also showed that DIOS treatment markedly downregulated the p-PI3K/PI3K, p-Akt/Akt, and p-mTOR/mTOR protein ratios, especially at the concentration of 40 µM DIOS (*p* < 0.05) (Figs. **5E**, **F**), confirming that DIOS was able to induce apoptosis and autophagy *via* suppressing the PI3K/Akt pathway in cutaneous melanoma cells.

### 3.6 DIOS Administration Induces Cell Apoptosis and Autophagy and Suppresses Tumor Growth *In vivo*

**Fig. (6):**
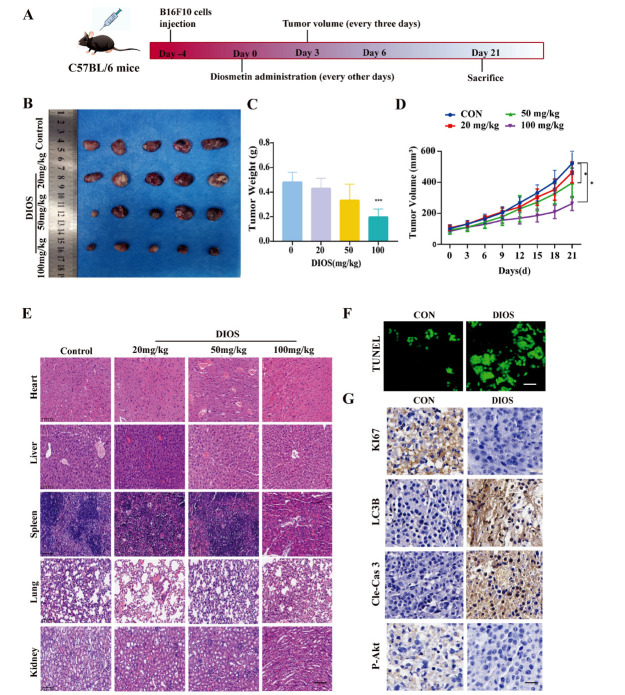
DIOS exerts an anti-melanoma effect *in vivo*. (**A**) Experimental design. (**B**) Morphological images of subcutaneous tumor xenografts from C57BL/6 mice. (**C**, **D**) Quantitative analysis of tumor weight and tumor volume (n=5). * *p* < 0.05, ** *p* < 0.01, *** *p* < 0.001 *vs*. the control group. (**E**) HE staining of the important viscera, original magnification: 20×, scale bar =100 μm. (**F**) TUNEL assay, original magnification: 20×, scale bar =100 μm. (**G**) Immunohistochemical detection of KI67, LC3B, Cleaved caspase 3, P-Akt expression. Original magnification: 40×, scale bar: 50 μm.

To evaluate the therapeutic potential of DIOS in a B16F10 syngeneic model, tumor-bearing mice were administered 20, 50, or 100 mg/kg of DIOS over a 21-day period (Fig. **6A**). The administration of 100 mg/kg of DIOS resulted in a notable inhibition of tumor growth in comparison to the control group (Fig. **6B**). Furthermore, the tumor weight in the 100 mg/kg DIOS treatment group was significantly lower than in the control group (*p* < 0.001), and both the 50 mg/kg and 100 mg/kg DIOS treatment groups exhibited significantly lower tumor volumes than the control group (*p* < 0.05) (Figs. **6C**, **D**). In addition, Fig. (**6E**) shows HE staining of viscera from DIOS-treated mice, showing no significant histological differences from those of the untreated group. These results showed that DIOS treatment had no obvious organ toxicity *in vivo*. Additionally, Tunel staining (Fig. **6F**) and immunohistochemistry for ki67 and Cleaved caspase 3 (Fig. **6G**) revealed a pronounced increase in the proportion of apoptotic cells following 100 mg/kg DIOS treatment. Positive immunohistochemistry results for LC3B (Fig. **6G**) further indicated that DIOS treatment enhanced cell autophagy *in vivo*. Moreover, 100 mg/kg DIOS treatment also led to the inhibition of Akt phosphorylation, as illustrated in Fig. (**6G**). These results indicate that DIOS is capable of inhibiting tumor growth, inducing cell apoptosis and autophagy, and inactivating the PI3K/Akt/mTOR pathway *in vivo*.

## 4 DISCUSSION

Natural compounds are widely recognized for their potent anti-cancer properties and low toxicity, making them highly effective in carcinoma treatment [38]. In China, the application of natural herbs in treating diseases spans thousands of years, marked by their remarkable therapeutic outcomes [22, 39, 40]. Diosmetin, a flavonoid found in medicinal herbs like citrus and Rosaceae plants [41, 42], has been demonstrated to mitigate myocardial apoptosis [43]. It has been suggested that DIOS showed anti-tumor properties and may be particularly promising as an anti-cancer agent in gastric cancer, helping to induce apoptosis and promoting protective autophagy [44]. Furthermore, it has been noted to hinder tumor advancement by triggering cell death in melanoma and suppressing angiogenesis, highlighting its promise as an anti-melanoma agent [45]. In this study, we aim to address critical gaps in understanding the mechanisms by which DIOS affects melanoma, specifically targeting its anti-cancer effects through apoptotic and autophagic pathways.

Apoptosis is a regulated process where cells deliberately induce their own death through gene regulation [46]. The Bcl-2 protein family is crucial in regulating apoptosis [47, 48]. Evidence suggests that certain drugs exhibit anti-cancer properties by enhancing drug-induced apoptosis [49]. Our study revealed that DIOS significantly suppressed the proliferation and migration of cutaneous melanoma cells, reduced the levels of Bcl-2, and increased the protein expressions of Cleaved caspase-9, Cleaved caspase-3, and Cleaved PARP. Additionally, *in vivo* experiments confirmed these results, showing a substantial reduction in tumor growth following DIOS treatment without showing apparent toxicity. With increasing concentration, DIOS further restrained melanoma cell proliferation and increased apoptosis. These results suggested that DIOS could inhibit proliferation and induce apoptosis of cutaneous melanoma cells, which were consistent with previous studies [35, 50, 51].

Autophagy is a fundamental cellular mechanism that conserves evolutionary traits and is important for the degradation of damaged components, clearing of intracellular waste, and upkeep of cellular equilibrium [52]. As previously mentioned in the literature review, LC3 is a critical component in autophagosome formation. Autophagy converts cytoplasmic LC3 (LC3-I) to its membrane form (LC3-II), which then binds to the inner membrane of the autophagosome until it is broken down inside the autophagolysosome [53, 54]. The protein p62, known for its ability to bind and assemble complexes with ubiquitinated proteins, is subsequently targeted for autolysosomal degradation together with LC3-II [55]. The formation and maturation of autophagosomes are facilitated by p62 and autophagy-related genes [56]. Compared with a benign nevus, Atg5 is frequently downregulated in primary melanoma, reducing basal autophagy as indicated by decreased LC3 [57, 58]. In the present study, DIOS significantly promoted the formation of autophagolysosome vacuole and enhanced LC3II/I and Atg5 expression while inhibiting p62 expression, indicating its impact on autophagy in melanoma cells. Both autophagy and apoptosis frequently occur simultaneously [59]. 3-MA, an autophagy-specific inhibitor, was applied to analyze the interplay between autophagy and apoptosis triggered by DIOS in melanoma cells [60]. Autophagy induction by chemotherapeutic and anti-tumor modalities has been extensively studied in various cancer models *in vitro* and *in vivo* [61, 62]. The dual roles of autophagy have been described as a “double-edged sword” in cell survival and proliferation [63, 64]. Inhibition of autophagy has been shown to result in apoptosis, thereby enhancing anti-tumor activity [65, 66]. Autophagic cell death, also known as type II programmed cell death, is increasingly accepted as essential for the anti-tumor activity of specific chemicals [49, 67]. Based on a current understanding, autophagy-targeted therapies are widely explored for skin cancer due to their great potential [68, 69]. Our study revealed that DIOS induced both apoptosis and autophagy in a dose-dependent manner. Inhibition of autophagy using 3-MA markedly reduced apoptotic cell numbers in cutaneous melanoma, suggesting that autophagy may inhibit cell survival and proliferation in response to diosmetin stress.

Previous research has identified a number of key molecules and signaling pathways that play a role in regulating autophagy [70, 71]. Cell proliferation, differentiation, and survival are significantly influenced by the PI3K/Akt/mTOR signaling pathway under normal and pathological conditions [72]. Drugs targeting this pathway have the potential to impair cancer cell survival mechanisms, thereby triggering apoptosis [73]. As a serine/threonine kinase, mTOR regulates various cellular signaling pathways and processes, including apoptosis and autophagy [74]. Previous studies have highlighted the importance of the PI3K/Akt/mTOR for DIOS-induced apoptosis and autophagy [75]. Notably, inhibiting the PI3K/Akt/mTOR in melanoma cells has shown a potential to suppress their growth and migration [76, 77]. In this study, our results demonstrate that DIOS uniquely targets the PI3K/Akt/mTOR pathway, which plays a pivotal role in regulating both apoptosis and autophagy. DIOS treatment led to decreased phosphorylation of PI3K, Akt, and mTOR in cutaneous melanoma cells, and immunohistochemistry revealed significantly higher p-Akt expression in the control group of tumor-bearing mice. Furthermore, GSEA analysis of transcriptome data highlighted significant enrichment of the PI3K/Akt pathway, supporting the conclusion that DIOS promotes apoptosis and autophagy through the inhibition of the PI3K/Akt/mTOR signaling pathway in cutaneous melanoma.

This study still has certain limitations. Firstly, we only used the subcutaneous transplantation model in C57BL/6 mice. Future research will involve developing an orthotopic xenograft and metastasis model for cutaneous melanoma, allowing for a deeper investigation of DIOS's mechanisms *in vivo*. Additionally, only a single molecular inhibitor was employed in this study to specifically disrupt autophagy. In forthcoming studies, we will further focus on research to improve the bioavailability of DIOS, for example, using materials such as nano-hydrogels, as well as researching the role and mechanism of anti-melanoma in combination with other targeted drugs.

## CONCLUSION

In summary, this study revealed that DIOS suppressed cell proliferation and triggered apoptosis and autophagy in both *in vitro* and *in vivo* models. Autophagy inhibition was found to degrade DIOS-induced apoptosis in cutaneous melanoma cells. Furthermore, the study suggested that DIOS could induce apoptosis and autophagy by restraining the PI3K/Akt/mTOR signaling pathway in cutaneous melanoma. These findings highlight the eligibility of DIOS to become a novel therapeutic agent for cutaneous melanoma.

## Data Availability

All data analyzed in this study are available from the corresponding author upon request.

## LIST OF ABBREVIATIONS

3-MA: 3-methyladenine
DEGs: Differentially Expressed Genes
DIOS: Diosmetin
DMSO: Dimethyl Sulfoxide
FBS: Fetal Bovine Serum
HE: Hematoxylin-eosin
MAPK/MMP: Mitogen-activated Protein Kinase/Matrix Metalloproteinases
TEM: Transmission Electron Microscopy
TGF-β: Transforming Growth Factor β
