# Supplementary material for: Effects of Citrus-derived Diosmetin on Melanoma: Induction of Apoptosis and Autophagy Mediated by PI3K/Akt/mTOR Pathway Inhibition
Source: Anticancer Agents Med Chem. 2025 Jan 21;25(13):921–33. doi: 10.2174/0118715206360266250115065234 (PMC12678984; doi:10.2174/0118715206360266250115065234)
Supplement: Supplementary file 1 — Supplementary material is available on the publisher's website along with the published article. [file ACAMC-25-13-921_SD1.pdf]

SUPPLEMENTARY MATERIAL

Effects of Citrus-derived Diosmetin on Melanoma: Induction of Apoptosis and Autophagy Mediated by PI3K/Akt/mTOR Pathway Inhibition

Jie Li[1,2,#], Mingyuan Xu[2,#], Nanhui Wu[2], Fei Wu[2], Jiashe Chen[2], Xiaoxiang Xu[2,*], Fei Tan[1,2,*] and Yeqiang Liu[1,2,*]

[1]Shanghai Skin Disease Clinical College, The Fifth Clinical Medical College, Anhui Medical University, Shanghai Skin Disease Hospital, Shanghai, 200443, China; [2]Shanghai Skin Disease Hospital, Tongji University School of Medicine, Shanghai, 200443, China

Supplementary Table 1. DIOS administration in control groups.

Group	Number				
Control	1	3	13	18	20
Dios 20mg/kg	6	15	14	7	9
Dios 50mg/kg	17	11	5	19	2
Dios100mg/kg	4	12	16	8	10
